# The impact of working conditions on sickness absence among older workers: a systematic review

**DOI:** 10.1007/s10433-026-00921-0

**Published:** 2026-04-25

**Authors:** Krisztina Gero, Christoph Benken, Nico Dragano

**Affiliations:** Medical Faculty and University Hospital, Centre for Health and Society, Institute of Medical Sociology, Heinrich Heine University Düsseldorf, Düsseldorf, Germany

**Keywords:** Healthy aging, Job demands, Sickness absence, Older workers

## Abstract

Population aging poses major challenges to the health and social systems of all countries. It also affects the labor force participation of older adults, shifting toward delayed retirement. However, older workers are more likely to face health issues, leading to productivity loss through sickness absence. To explore factors associated with illness-related absence while considering the significant amount of time spent at work, we conducted a systematic review assessing the effects of physical and psychosocial working conditions on sickness absence among older workers aged ≥ 50 years. A systematic search was conducted in the MEDLINE, CINAHL Ultimate, PsycInfo, Scopus, and Web of Science databases on December 3, 2025. Additionally, a forward and backward reference search was performed. Research that focused on older working adults was scarce. In total, 24 articles were included. Results showed that high physical job demands, physical job hazards, high psychosocial job demands, low job control, low rewards, effort–reward imbalance, low collegial and high managerial social support, low procedural justice, medium role clarity, high role conflicts, skill variety, certain work-time-related characteristics (e.g., shift work, long working hours, and non-flexible work hours), and—among women—sexual violence were all associated with a higher risk of sickness absence among older workers. However, some of the presented findings were based on single studies. We identified significant work-related factors for designing interventions and a supportive environment to reduce sick leave in elder workers. Nevertheless, further age-stratified research is necessary to strengthen the evidence and accommodate the needs of the aging, economically active population.

**Supplementary Information:**

The online version contains supplementary material available at 10.1007/s10433-026-00921-0.

## Introduction

Global life expectancy has continuously increased during the past three decades. As a result, the share of the world’s population aged 60 years and over reached one billion in 2020 and is expected to double (2.1 billion) by 2050 (World Health Organization 2024a). Population aging poses major challenges to the health and social systems of all countries. It also affects the labor force participation of older adults. Accordingly, retirement policies started to reflect this demographic shift, supporting and encouraging delayed retirement (National Bureau of Economic Research 2019; Dai et al. 2022). However, older workers are more likely to face health issues, potentially compromising their ability to continue working.

Poor mental and physical health can lead to work-related outcomes such as temporary sickness absence and—eventually—to early workforce exit (OECD 2023). Absence from work is a crucial component of recovery during illness, providing individuals with the necessary time to rest, recuperate, and prevent the spread of contagious diseases within the workplace. Sick leave has been shown to support mental and physical health, reduce the risk of long-term disability, and facilitate a sustainable return to work (Cornelius et al. 2011; Villotti et al. 2024). However, when sick leave is associated with potentially avoidable work hazards, it becomes a serious concern for the labor market, potentially diminishing the productive capacity of both individuals and organizations. Addressing workplace conditions and implementing preventive measures are essential to minimize unnecessary sick leave and maintain workforce productivity (OECD 2023). In the European Union, absenteeism due to illness reached an average of 14 days per employee per year in 2019 (World Health Organization 2024b). In addition, during the same year, the average sickness absence rate was 3.2% among European older workers aged 55 to 64 years. This rate was significantly higher than among their younger counterparts (15–29 years: 1.3%; 30–54 years: 2%), also representing substantial direct and indirect illness-related costs (OECD 2023). Thus, exploring preventable factors associated with sickness absence—with a particular focus on potentially vulnerable older adults—is imperative to developing relevant interventions for mitigating avoidable sick leave and providing the right environment for a longer and healthier working life.

However, while it is well-established that the working environment affects health, no reports have assessed the published literature to gain an overview of specific working conditions linked to sickness-related absence among older workers. Such a look at studies that have specifically examined older employees appears warranted, as it cannot be assumed that findings from younger age groups or from studies covering the entire age spectrum can be readily transferred to older people. In particular, it should be noted that both the type and frequency of exposure to work-related hazards, as well as susceptibility to their health effects, change with age (Crawford et al. 2010; Hanvold et al. 2019; Wegman & McGee 2004). Examples include the frequently observed increase in autonomy with greater seniority, as well as increased vulnerability to physical workloads such as heavy lifting or working in awkward postures with increasing age. Therefore, we conducted a systematic review to examine and summarize the existing evidence regarding the associations between working conditions and sickness absence among working adults aged 50 years and over. We aimed to explore the effects of physical and psychosocial work-related factors—without a strict definition of what is considered part of the working environment—to identify knowledge gaps and inform future research, policy, and practice.

## Methods

A systematic review was conducted in accordance with the Preferred Reporting Items for Systematic Reviews and Meta-Analyses (PRISMA) guideline (Page et al. 2021). After performing a preliminary literature search, piloting the study selection process, and starting formal screening of search results against eligibility criteria, a protocol was registered in the International Prospective Register of Systematic Reviews (PROSPERO ID: CRD42024605280).

### Inclusion/exclusion criteria

Peer-reviewed original articles published in international journals assessing the associations between working conditions and sickness absence were examined. All exposures related to the physical and psychosocial working environment were regarded as eligible; strict definitions were not made. Sickness absence was defined as being absent from work for a short- or long-term period due to any kind of sickness or ill health. While we acknowledge the importance of a life course perspective regarding exposure to health risks, to identify factors affecting specifically older workers, the target population was limited to those aged 50 years and older.

Observational studies published in English or German were evaluated for inclusion. However, reviews and meta-analyses were excluded along with intervention studies, case reports, qualitative studies, conference abstracts, comments, letters, editorials, government-commissioned reports, and “grey literature.”

### Search strategy

A systematic search was conducted among abstracts and titles in the MEDLINE (EBSCOhost), CINAHL Ultimate (EBSCOhost), PsycInfo (EBSCOhost), Scopus, and Web of Science databases on the 29th of August 2024, using the following search string: (workplace* OR ("human resource" N1 management) OR (work* N1 management) OR (work* N2 environment*) OR (work* N2 condition*) OR (work* N1 organization*)) AND (sick* absen* OR absentee* OR presentee* OR "long-term ill*" OR "sick* day*" OR "sick* leave*") AND (old* OR elderly OR senior* OR aged OR aging OR aging).

To ensure that the review remained up to date considering the time elapsed during the publication process, an additional search was conducted on December 3, 2025. In this update, the search strategy was refined by adding the following terms to better capture work-time-related exposures, in response to the limited number of relevant studies identified in the initial search: (work* N2 schedule) OR (long* N2 "work* hours") OR (work* N2 location*).

Forward and backward reference search of the included articles was also performed via Web of Science.

### Study selection and data extraction

Duplicates of titles and abstracts were identified using Rayyan software. They were resolved manually and screened according to the inclusion and exclusion criteria. Finally, the full texts of the selected studies were evaluated for final inclusion. Two reviewers independently conducted the screening process. Any conflicts regarding the eligibility of publications for inclusion were resolved through discussions between the reviewers. Data were extracted from the included articles by two authors, with the main reviewer handling 75% of the extraction and the second reviewer handling 25%. Additionally, both reviewers quality-checked the data extracted by the other. Data synthesis included the following information: year of publication, author list, article title, study location, study population, mean age and age range of the study population, study design, outcome definitions, exposure definitions, and results summary. Study quality was assessed using design-specific tools. Cohort studies were appraised using the Newcastle–Ottawa Scale (NOS), with particular emphasis on adjustment for baseline health or prior sickness absence as key confounders (Wells et al. 2025). Cross-sectional studies were evaluated using an adapted NOS for cross-sectional designs (Carra et al. 2025). The included case-crossover study was assessed using the Joanna Briggs Institute (JBI) case–control checklist adapted for within-person designs (JBI 2020). Overall study quality was classified based on established criteria and applied consistently across study designs.

## Results

### Screening process

**Fig. 1:**
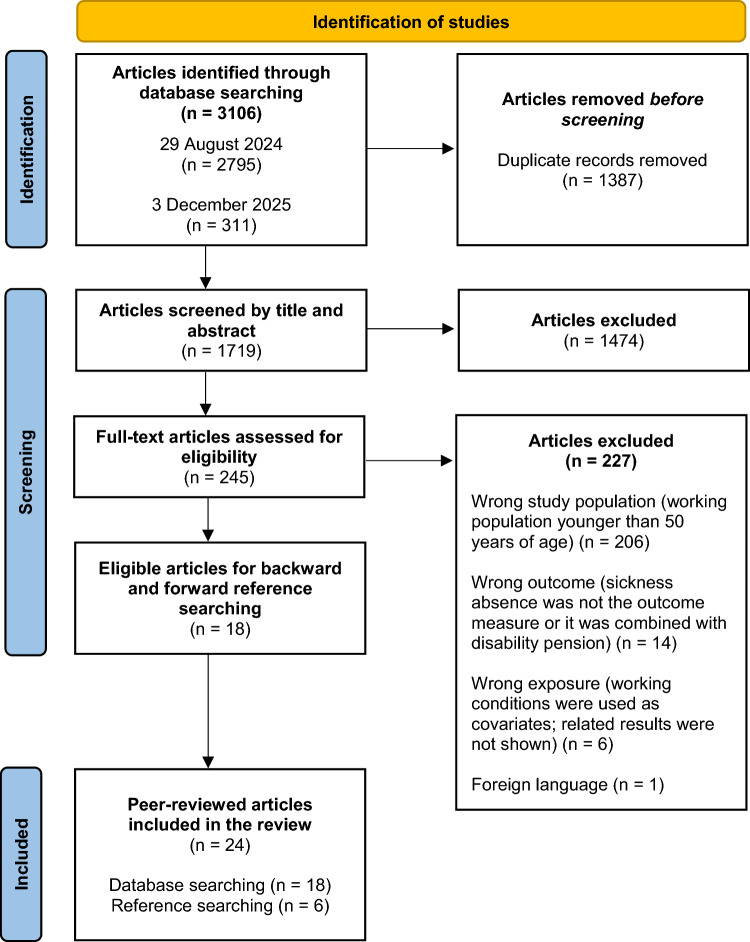
PRISMA flowchart showing the study selection process

 As illustrated in Fig. 1, we identified 2795 potentially relevant records through a database search conducted on the 29th of August 2024 in MEDLINE (EBSCOhost platform; *n* = 484), CINAHL Ultimate (EBSCOhost platform; *n* = 228), PsycInfo (EBSCOhost platform; *n* = 221), Web of Science (*n* = 1214), and Scopus (*n* = 648). After removing duplicates (*n* = 1387), the title and abstract of 1408 articles remained for screening. Following an updated search conducted on the 3rd of December 2025, an additional 311 records were identified and screened at the title and abstract level, resulting in a total of 1719 records assessed for eligibility. Overall, 245 studies were selected for full-text screening, including 43 additional articles identified during the update search, resulting in 18 articles qualifying for the review. During the final screening stage, the main reasons for exclusion were: 1. wrong target population (wide age range, age-stratified results were not presented for those aged 50 years or older; *n* = 206), 2. wrong outcome (sickness absence was not the outcome measure or it was combined with disability pension; *n* = 14), and 3. wrong exposure (working conditions were used as covariates, related results were not shown; *n* = 6). A backward and forward reference search was conducted based on the included studies, resulting in six additional records being added to the final review.

### Study characteristics

The majority of the included peer-reviewed studies were conducted in Denmark (*n* = 10), followed by Finland (*n* = 5), Sweden (*n* = 3), France (*n* = 3), Germany (*n* = 2), Iceland (*n* = 1), and Norway (*n* = 1). The study design of the articles was mainly prospective cohort (*n* = 18), with an additional *n* = 2 prospective panel, *n* = 3 cross-sectional studies, and *n* = 1 case-crossover study. Using the Newcastle–Ottawa Scale and the adapted JBI checklist, 22 studies were rated as good, one study as fair, and one study as poor quality. The study rated as poor quality showed adequate statistical modeling but was limited by potential attrition bias and reliance on self-reported outcome measures (Piszczek and Pimputkar 2021). The study rated as fair quality was characterized by acceptable, but not gold-standard, exposure and outcome measurements and did not adjust for health-related covariates (Bouville et al. 2018). Overall, most included studies demonstrated adequate methodological quality.

Supplementary Tables 1 to 4 summarize the main study characteristics and findings (Online Resource 1). Due to the heterogeneity of the exposure variables, the findings were categorized into three main topics: (1) physical working conditions, (2) psychosocial working conditions, and (3) work-time-related exposures.

### Physical working conditions

Several included articles focused on work-related physical requirements, as well as the physical work environment, and their effects on health. Physically demanding work, often assessed as a composite index, was consistently associated with short- and long-term sickness absences among older workers (Sundstrup et al. 2018a; Pedersen et al. 2020, 2022; Andersen et al. 2021). This composite generally includes factors such as heavy lifting (typically at least 15 kg) (Sundstrup et al. 2017, 2018a; Oliv et al. 2019; Bláfoss et al. 2023), pulling or pushing of heavy burdens (Sundstrup et al. 2018a), repetitive movements (Sundstrup et al. 2018a; Oliv et al. 2019), and certain work postures (Siukola et al. 2011), such as kneeling (Sundstrup et al. 2017) or working with twisted/bent back (Sundstrup et al. 2018a; Oliv et al. 2019), all of which were also independently linked to a significantly higher risk of sickness absence. Exposure to vibrations, including hand-tool vibrations and whole-body vibrations, was significantly linked to long-term sickness absence among older workers in Denmark who had 0 to 10 years of exposure (Sundstrup et al. 2017, 2018a). However, this association was not observed among individuals with neck or upper back pain in Sweden (Oliv et al. 2019).

A few studies also focused on the health effects of selected job hazards in the physical working environment. Specifically, exposure to noise, dust (cement, demolitions, mineral fibers, wood, animals or plants), diesel fumes, toxic substances, and welding smoke was linked to a higher risk of register-based long-term sickness absence in Denmark (Sundstrup et al. 2018a), while exposure to heating, ventilation, and air-conditioning was associated with self-reported sickness absence in France (Preziosi et al. 2004).

However, in a study conducted in Finland, researchers found no significant links between changes in drought, noise, indoor climate, lighting, heat, or exposure to cold after four years and the number of days of sickness absence (Siukola et al. 2011).

### Psychosocial working conditions

High job demands (Bouville et al. 2018; Sundstrup et al. 2018b; Wang et al. 2022; Shiri et al. 2024) and low job control (Oliv et al. 2019; Wang et al. 2022; Shiri et al. 2024) as composite factors, as well as autonomy (Bouville et al. 2018) and low influence (Sundstrup et al. 2018b), were significantly associated with short- and long-term sickness absences. However, results pertaining to the demand–control ratio were not statistically significant among older workers (Sundstrup et al. 2018b; Farrants et al. 2022; Shiri et al. 2024). Also, while high emotional and cognitive job demands were significantly associated with a higher risk of long-term sickness absence, this did not hold for quantitative demands and work pace (Sundstrup et al. 2018b). On the other hand, high work effort (Götz et al. 2018), low job rewards (Götz et al. 2018), and effort–reward imbalance (Götz et al. 2018; Sundstrup et al. 2018b) were all linked to a higher risk of sickness absence.

In addition, findings from two Finnish studies suggest that changes in certain psychosocial work factors over time may not be strongly associated with illness-related absence among older workers. One study found that reductions in work effort and effort–reward imbalance, as well as improvements in job rewards or work-time control, did not significantly correlate with the likelihood of illness-related absences lasting more than ten consecutive workdays (Shiri et al. 2024). Similarly, another study examining changes in sickness absence days over a four-year period found no significant associations with shifts in various organizational and motivational factors, including team spirit and reactivity, incentive systems, extrinsic incentives, incentive and participative leadership, task and goal systems, task value, and opportunities to influence one’s work (Siukola et al. 2011).

As for social support, the related results were also mixed. A survey based on single questions showed no significant relationship between social support from colleagues or supervisors and long-term sickness absence in Denmark (Sundstrup et al. 2018b). In contrast, studies using more complex measures (i.e., a Job Exposure Matrix or four items from Karasek’s Job Content Questionnaire) reported significant associations, linking higher at-work (Wang et al. 2022) or supervisor support (Bouville et al. 2018) to a higher risk and higher colleague support (Bouville et al. 2018) to a lower risk of sickness absence among older workers.

Other assessed psychosocial risk factors that were significantly associated with sickness absence included low procedural justice (Tenhiälä et al. 2013), medium (but not low) role clarity (Sundstrup et al. 2018b), high role conflicts (Sundstrup et al. 2018b), skill variety (Bouville et al. 2018), generic measures of work-related stress (Pedersen et al. 2021, 2024), and—among women—sexual violence (when exposed in current/former working environment, or both) (Jonsdottir et al. 2024).

### Work-time-related exposures

Five articles examined associations between work-time-related exposures and sickness absence among older workers. One study among male manual workers aged 50–59 years in the French private sector found no significant associations between irregular or 24-h shift work and sickness absence compared with regular schedules (Afsa and Givord 2014).

A longitudinal multilevel study among Norwegian hospital employees reported higher predicted probabilities of both short-term (1–8 days) and long-term (≥ 9 days) sickness absence among workers engaged in rotating day–evening and day–evening–night shifts compared with those working fixed day shifts at age 50 years, with stronger associations observed at age 60 (Bernstrøm and Houkes 2020).

A register-based cohort study of Danish and Finnish nurses aged 50–67 years reported that in the Danish cohort, frequent evening and night shifts (> 50 shifts per year), very long work weeks (> 48 h), and consecutive night shifts (≥ 5) were associated with an increased risk of first-time long-term sickness absence (≥ 30 consecutive days), whereas a higher number of day shifts (> 100 shifts per year) were associated with a lower risk. In the Finnish cohort, long shifts (≥ 9 to < 12 h, > 50 shifts per year) and long work weeks (> 40 h, 1–50 per year) were associated with an increased risk, while day shifts, evening shifts, night shifts, very long shifts (≥ 12 h), quick returns, very long work weeks (> 48 h), and consecutive night shifts were not significantly associated with long-term sickness absence (Larsen et al. 2020).

A case-crossover study among Finnish public sector employees aged 55 years and older found that higher weekly working hours, a greater proportion of weeks exceeding 40 h, and a higher proportion of morning shifts were associated with an increased risk of short-term sickness absence (1–3 days), whereas daily working hours, long shifts (≥ 12 h), evening or night work, and measures of shift intensity showed no significant associations (Ropponen et al. 2020).

Finally, a prospective panel study among German employees aged 50–65 years found that flexible working hours were associated with lower self-reported sick day use, with statistically significant effects observed from age 51 onwards (Piszczek and Pimputkar 2021).

### Heterogeneity of measures and methods

The included studies exhibit considerable methodological heterogeneity. This relates to the populations included, the operationalization of outcomes and exposures, as well as statistical modeling approaches. For example, sickness absence was assessed using different methods (register data, self-reports), and, in some cases, different cut-off points were applied (see Supplementary Tables 1–4).

It is important to mention that several of the included studies accounted for previous (baseline) health by adjusting their statistical evaluations by covariates, namely self-rated health (Götz et al. 2018; Shiri et al. 2024), depressive symptoms (Pedersen et al. 2020, 2022; Bláfoss et al. 2023), psychological distress (Tenhiala et al. 2013), previous treatment for various diseases (Pedersen et al. 2022), chronic diseases (Sundstrup et al. 2018a, 2018b) or (long-term) sickness absence (Siukola et al. 2011; Sundstrup et al. 2018a, 2018b; Bernstrom and Houkes 2020; Farrants et al. 2022; Wang et al. 2022). Furthermore, health-related factors such as BMI, obesity, smoking, alcohol consumption, physical activity, and sleep measures were also considered (Preziosi et al. 2004; Tenhiala et al. 2013; Sundstrup et al. 2017; Sundstrup et al. 2018a, 2018b; Pedersen et al. 2021; Andersen et al. 2021; Bláfoss et al. 2023; Pedersen et al. 2024; Shiri et al. 2024). However, there were still some studies not accounting for any relevant health-related factors (Afsa and Givord 2014; Bouville et al. 2018; Oliv et al. 2019; Ropponen et al. 2020; Pedersen et al. 2020; Piszczek and Pimputkar 2021; Jonsdottir et al. 2024).

## Discussion

This review found that high physical job demands and hazardous physical work environments were consistently associated with sickness absence among older workers, regardless of the measure used (Preziosi et al. 2004; Sundstrup et al. 2017, 2018a; Oliv et al. 2019; Pedersen et al. 2020, 2022; Andersen et al. 2021; Bláfoss et al. 2023). However, findings related to the psychosocial working environment were more complex, occasionally yielding unexpected results that require further clarification. While high work effort (Götz et al. 2018), low job rewards (Götz et al. 2018), and effort–reward imbalance (Götz et al. 2018; Sundstrup et al. 2018b) have repeatedly been tied to a higher risk of sickness absence, not all psychosocial indicators showed statistically robust effects. For instance, although high job demands (Bouville et al. 2018; Sundstrup et al. 2018b; Wang et al. 2022; Shiri et al. 2024) and low job control (Oliv et al. 2019; Wang et al. 2022; Shiri et al. 2024) were each linked to increased sickness absence risk, the associations for the demand/control ratio were not statistically significant (Sundstrup et al. 2018b; Farrants et al. 2022). Still, regarding the latter, it has to be noted that one of the two related studies used a psychosocial job exposure matrix to determine demand-control status (Farrants et al. 2022)—potentially ignoring individual differences within the same occupation—while the other focused on “work organization and job contents,” including “possibilities for development,” to define job control (Sundstrup et al. 2018b). Other approaches should be explored as well before drawing strong conclusions. At this point, it is worth noting once again the considerable degree of heterogeneity in the methods used in the included studies (e.g., with regard to outcome definition or exposure measurement), which makes comparisons between them significantly more difficult—a circumstance that applies to all results considered in this manuscript. To add to these issues, some included studies lack the consideration of previous (baseline) health or health-related factors as potential confounders or effect modifiers. Previous research points to the relevance of baseline health for the association between the effects of pension reforms on the length of working lives, morbidity, and sickness absence. But since most studies took such factors into account during their analysis, this issue might pose only a limitation to the generalizability of some of the findings.

In addition, both articles assessed long-term sickness absence as the outcome (> 30 calendar days (Sundstrup et al. 2018b) or > 183 net days (Farrants et al. 2022)). Therefore, the associations between demand-control status and short-term sickness absence remained unclarified. Similarly, only one study analyzed the effects of specific job demand items such as “quantitative demands” and “work pace” among older workers, necessitating further evaluation (Sundstrup et al. 2018b).

Evidence was also scarce regarding the effects of changes in psychosocial work factors on sickness absence. One of the two relevant Finnish studies focused on differences in job demands, job control, the demand–control ratio, work effort, job rewards, effort–reward imbalance, and work-time control between 2018 and 2020 among workers aged 50 years and older (Shiri et al. 2024). Although a reduction in job demands was linked to a lower risk of long-term sickness absence, most other changes showed no significant associations (Shiri et al. 2024). A potential explanation is that the two-year time period may have been too short for positive changes in the work environment to translate into measurable health effects. Furthermore, the precise timing of exposure change within the assessment period was unknown, limiting causal inference (Shiri et al. 2024). The second Finnish study, which also investigated changes in psychosocial working conditions over time in relation to sickness absence days, similarly found no significant associations (Siukola et al. 2011). Specifically, changes in organizational and motivational dimensions—such as team spirit and reactivity, incentive systems, extrinsic incentives, incentive and participative leadership, task and goal structures, task value, and opportunities to influence one’s work—were not linked to changes in sickness absence (Siukola et al. 2011). These findings suggest that even when workplace conditions improve in theory, the impact on actual health-related outcomes may be limited or delayed, especially in older populations with increased vulnerabilities due to previous exposures and preexisting disease. It is also possible that more nuanced or sustained improvements are needed before meaningful reductions in sickness absence become observable.

Findings on social support were also mixed, calling for further research. Measurement approaches differed across studies, suggesting that the use of single-item questions may not be sufficient to detect significant associations between workplace social support and sickness absence (Sundstrup et al. 2018b). Alternatively, the specific questions asked may not have captured the most relevant dimensions of social support among older workers. Furthermore, a cross-sectional study from France reported that while high supervisor support was linked to a lower risk of sickness absence among younger workers, the opposite was observed among older employees (Bouville et al. 2018). One possible explanation is that supportive supervisors may be more willing to accommodate the health-related needs of older employees by encouraging appropriate use of sick leave or by reducing informal pressure to attend work when unwell. This may result in higher recorded sickness absence but potentially lower levels of presenteeism. However, these findings should be confirmed in longitudinal studies conducted among other worker populations aged 50 years or older.

Five included studies examined the impact of work-time-related exposures on sickness absence among older workers. One study reported no significant associations between 24-h shift work or irregular schedules (compared to regular schedules) and sickness absence among male manual workers aged 50 to 59 years in the French private sector (Afsa and Givord 2014). These null findings may be partly explained by sample-specific factors. The study population likely represented a highly selected group of older workers who remained employed under physically and socially demanding conditions, possibly reflecting a “healthy worker effect” (Afsa and Givord 2014). In addition, financial incentives, such as wage premiums, may also have mitigated adverse effects in this group (Afsa and Givord 2014).

In contrast, several studies reported associations between specific working time characteristics and sickness absence. Rotating shift systems were linked to higher probabilities of both short-term and long-term sickness absence among older hospital employees, with stronger associations observed at higher ages (Bernstrøm and Houkes 2020). Among Danish and Finnish nurses, frequent evening and night shifts, very long work weeks, and consecutive night shifts were associated with increased risks of long-term sickness absence, although findings differed somewhat between countries (Larsen et al. 2020). A case-crossover study further showed that higher weekly working hours and a greater proportion of long work weeks were associated with an increased risk of short-term sickness absence among employees aged 55 years and older, although several other indicators of shift intensity and long shifts were not consistently associated with absence (Ropponen et al. 2020). In addition, a German panel study found that flexible working hours were associated with lower sick day use among employees aged 51 years and older, suggesting that work-time autonomy may play a protective role in later working life (Piszczek and Pimputkar 2021).

Overall, the evidence indicates that certain forms of shift work and extended working hours are associated with increased sickness absence among older workers, whereas work-time flexibility may reduce absence. However, findings varied across occupational contexts and exposure definitions, highlighting the need for further age-specific research on working time arrangements.

There are some limitations to the current review that should be taken into consideration. We may have inadvertently missed relevant peer-reviewed articles during the screening process. In addition, only articles published in English or German were considered. Consequently, the included publications may not fully represent the older working populations of non-English or German-speaking countries. However, only one potentially relevant article published in Polish was identified during the screening process. As the full text was not accessible, it could not be evaluated for inclusion. While this may have resulted in the omission of some evidence, the impact on the overall conclusions is likely to be limited.

Only a few articles presented sex-stratified findings. As such, potential sex differences in the effects of working conditions on sickness absence could not be assessed. Moreover, the low number of identified studies from just a small number of countries limits the generalizability of the findings of this review. In this context, it should be noted that the search strategy limited to a specific age range likely led to the exclusion of important studies on general predictors of work disability, insofar as these examined younger populations or employed an age-unspecific presentation of results. The restricted scope focusing on older individuals must be taken into account when interpreting the findings, and, where appropriate, additional evidence from general studies (e.g., Fisker et al. 2022; Margheritti et al. 2026) should be considered. Finally, it would have been of interest to contrast the findings summarized here with results from comparable studies in younger populations. While this was not part of the research question, it would represent a worthwhile topic for future syntheses.

Although this is not a direct limitation of this review, we would also like to point out two additional sources of uncertainty in the interpretation of the studies summarized here. First, selection effects should be considered. In particular, a potential healthy worker bias should be mentioned, which may be more pronounced in older than in younger employees (e.g., better opportunities for older employees with preexisting conditions to retire early from working life). Second, it should be borne in mind that focusing solely on sickness absence may underestimate the potential impact of working conditions on health, for example because employees may not take sick leave despite illness (presenteeism).

## Conclusion

This systematic review identified physical and psychosocial work factors that should be considered when designing interventions and a supportive environment for healthy aging at work. The substantial health effects of physical job demands, physical job hazards, and domains of the psychosocial work environment—such as job control, rewards, effort–reward imbalance, social support, and skill discretion—as well as work-time-related characteristics, including shift work, long work weeks, and flexible working hours—among others—cannot be overlooked. However, studies specifically focusing on older workers remain scarce, even though certain working conditions may affect them differently than younger employees. Moreover, while multiple risk factors were identified, some findings were based on single studies. Therefore, further age-stratified research is needed to inform targeted solutions that address the specific needs of the aging, economically active population. In particular, studies with broader age ranges should be encouraged to present stratified estimates for different age groups in order to allow for a direct assessment of age-dependent effects. In addition, it would be helpful if methodological heterogeneity could be reduced in future studies.

## Supplementary Information

Below is the link to the electronic supplementary material.

Supplementary file1 (DOCX 41 KB)

## Data Availability

No datasets were generated or analyzed during the current study.
