# Supplementary material for: The impact of working conditions on sickness absence among older workers: a systematic review
Source: Eur J Ageing. 2026 Apr 25;23(1):21. doi: 10.1007/s10433-026-00921-0 (PMC13110265; doi:10.1007/s10433-026-00921-0)
Supplement: Supplementary file 1 — Supplementary file1 (DOCX 41 KB) [file 10433_2026_921_MOESM1_ESM.docx]

**The impact of working conditions on sickness absence among older workers: A systematic review**

European Journal of Ageing

Krisztina Gerő, PhD^1^, Christoph Benken^1^, and Nico Dragano, PhD^1^

^1^Institute of Medical Sociology, Centre for Health and Society, Medical Faculty and University Hospital, Heinrich Heine University Düsseldorf, Düsseldorf, Germany

E-mail address of the corresponding author: [Christoph.Benken@med.uni-duesseldorf.de](mailto:Christoph.Benken@med.uni-duesseldorf.de)

**Supplementary Table 1.** Main characteristics of studies assessing the impact of working conditions on sickness absence

| **Author** | **Study location** | **Study population** | **Mean age (SD)** | **Age range (years)** | **Study design** | **Quality assessment (NOS)** |
| --- | --- | --- | --- | --- | --- | --- |
| Jonsdottir et al. 2024 | Iceland | Women participants in the Icelandic Stress-and-Gene-Analysis (SAGA) cohort | N/A (Age-stratified) | 55–69 | Cross-sectional study | Good |
| Shiri et al. 2024 | Finland | Employees from 11 municipalities from the Finnish Public Sector study | N/A (Age-stratified) | 50+ | Prospective cohort study | Good |
| Pedersen et al. 2024 | Denmark | Work Environment and Health in Denmark (WEHD) study linked with the Danish labor market accountant (LMA), work absences (RoWA), education, emigration and immigration, and the death registers | N/A (Age-stratified) | 50–64 | Prospective cohort study | Good |
| Bláfoss et al. 2023 | Denmark | Currently employed wage earners with lifting tasks at work without incident long-term sickness absence (LTSA) 52 weeks preceding the questionnaire reply, drawn from a probability sample of Danish citizens who worked for at least 35 hrs/month and earned ≥3000 DKK (approximately €400)/month for the past three months – Work Environment and Health in Denmark (WEHD) linked to the Danish Register for Evaluation of Marginalisation (DREAM) | N/A (Age-stratified) | 50–64 | Prospective cohort study | Good |
| Farrants et al. 2022 | Sweden | Swedish Work Environment survey and the Longitudinal integration database for health insurance and labour market studies (LISA) | N/A | 55–64 | Prospective cohort study | Good |
| Pedersen et al. 2022 | Denmark | Work Environment and Health in Denmark (WEHD) survey linked with the Danish Labor Market Accountant Register (LMAR), Register of Work Absences (RoWA), the Education Register, Emigration and Immigration Register, and the Death Register | Men: 56.6  Women: 56.2 | 50–64 | Prospective cohort study | Good |
| Wang et al. 2022 | Sweden | Swedish Twin project of Disability pension and Sickness absence (STODS) | N/A (Age-stratified) | 56–64 | Prospective cohort study | Good |
| Andersen et al. 2021 | Denmark | Work Environment and Health in Denmark study (WEHD) / Danish Register for Evaluation of Marginalization (DREAM) | N/A (Age-stratified) | 60–64 | Prospective cohort study | Good |
| Pedersen et al. 2021 | Denmark | Work Environment and Health in Denmark (WEHD) survey linked with the Danish Labor Market Accountant Register (LMAR), Register of Work Absences (RoWA), Employment Register, Education Register, Emigration and Immigration Register, and the Death Register | N/A (Age-stratified) | 50–59 | Prospective cohort study | Good |
| Piszczek et al. 2021 | Germany | Linked Personnel Panel (LPP): survey contains data only from employers (and a sample of their employees) – representative of employers of this size | N/A (Age-specific) | 50–65 | Prospective panel study | Poor |
| Bernstrøm and Houkes 2020 | Norway | Full-time working employees from a large Norwegian hospital – multiple locations (2012-2016) | N/A (Age-specific) | 50–50  60–60 | Prospective cohort study | Good |
| Larsen et al. 2020 | Denmark, Finland | Danish Working Hour Database / Finnish Public Sector Study | N/A (Age-stratified) | 50–67 | Prospective cohort study | Good |
| Pedersen et al. 2020 | Denmark | Statistics Denmark / Danish Register for Evaluation of Marginalisation (DREAM) | N/A (Age-specific) | 50–50 | Prospective cohort study | Good |
| Ropponen et al. 2020 | Finland | Finnish Public Sector study | N/A (Age-stratified) | 55+ | Case-crossover study | Good (JBI) |
| Oliv et al. 2019 | Sweden | Swedish Work Environment survey and the Longitudinal integration database for health insurance and labour market studies (LISA) | N/A (Age-stratified) | 50–64 | Prospective cohort study | Good |
| Bouville et al. 2018 | France | SUrveillance MEdicale des Risques professionnels (SUMER – Medical monitoring of occupational risks) 2003 survey | N/A (Age-specific) | 49–49 | Cross-sectional study | Fair |
| Götz et al. 2018 | Germany | Full-time employed workers from the German socio-economic panel (GSOEP) who were not permanently sick | N/A (Age-stratified) | 58–65 | Prospective panel study | Good |
| Sundstrup et al. 2018 | Denmark | Copenhagen Aging and Midlife Biobank (CAMB) cohort (employed wage earners at baseline) / Danish Register for Evaluation of Marginalization (DREAM) | 54.3 (3.8) | 49–63 | Prospective cohort study | Good |
| Sundstrup et al. 2018 | Denmark | Copenhagen Aging and Midlife Biobank (CAMB) cohort (employed wage earners at baseline) / Danish Register for Evaluation of Marginalization (DREAM) | 54.3 (3.8) | 49–63 | Prospective cohort study | Good |
| Sundstrup et al. 2017 | Denmark | Copenhagen Aging and Midlife Biobank (CAMB) cohort (individuals who were on the labor market at baseline) / Danish Register for Evaluation of Marginalization (DREAM) | 54.3 (3.8) | 49–63 | Prospective cohort study | Good |
| Afsa and Givord 2014 | France | Male manual workers in the private sector from the Labor Force Survey (LFS) | N/A (Age-stratified) | 50–59 | Pooled cross-sectional study | Good |
| Tenhiala et al. 2013 | Finland | Public sector employees from ten towns and 21 public hospitals in Finland | N/A (Age-stratified) | 56–67 | Prospective cohort study | Good |
| Siukola et al. 2011 | Finland | All employees – Finnish Food Industry Company | N/A (Age-stratified) | 50–62 | Prospective cohort study | Good |
| Preziosi et al. 2004 | France | Professionally active women from the SU.VI.MAX cohort | Exposed: 53.0 (13.4)  Non-exposed: 54.9 (4.2) | 49–65 | Prospective cohort study | Good |

**Supplementary Table 2.** Summary of main results by exposure to psychosocial working conditions within each included article

| **Author** | **Outcome definition** | **Exposure** | **Exposure definition** | **Results summary** |
| --- | --- | --- | --- | --- |
| Jonsdottir et al. 2024 | Sick leave from work: "How often have you been on sick leave for 15 or more consecutive days in the past 12 months?" (never, once, or more than once – converted into binary variable: never, 0; once or more, 1) | sexual violence | Sexual violence: "Have you experienced sexual harassment or violence in your working environment?" (no; yes, in my current working environment; yes, in my former working environment; and yes, in both my current and former working environment – lifetime exposure included previous, current, or both; ever vs. never) | Significant: women exposed to workplace sexual violence (ever; previous; current & both) were more likely than non-exposed to have taken one or more periods of sick leave (≥15 consecutive days) from work in the past year |
| Shiri et al. 2024 | Registered sickness absence: at least one episode of illness-related absence lasting more than 10 consecutive workdays – obtained from the employers’ records | job demands | Job demands: five items | Significant: reduction in job demands was associated with a lower risk of sickness absence |
| Shiri et al. 2024 | Registered sickness absence: at least one episode of illness-related absence lasting more than 10 consecutive workdays – obtained from the employers’ records | job control | Job control: nine items | Marginally significant: improvement in job control was marginally significantly associated with a lower risk of sickness absence |
| Shiri et al. 2024 | Registered sickness absence: at least one episode of illness-related absence lasting more than 10 consecutive workdays – obtained from the employers’ records | demand/control status | Job demand–control ratio (i.e., job strain): demands score divided by the control score multiplied by 0.556 (correction factor) | Non-significant: reduction in job demand–control ratio was not significantly associated with sickness absence |
| Shiri et al. 2024 | Registered sickness absence: at least one episode of illness-related absence lasting more than 10 consecutive workdays – obtained from the employers’ records | work effort | Effort at work: one item – "how much do you invest your talents and resources in the work" | Non-significant: reduction in work effort was not significantly associated with sickness absence |
| Shiri et al. 2024 | Registered sickness absence: at least one episode of illness-related absence lasting more than 10 consecutive workdays – obtained from the employers’ records | job rewards | Rewards: three items – how much the participants received compensation for their work in terms of (i) income, employment benefits and other monetary rewards, (ii) recognition and appreciation, and (iii) personal satisfaction | Non-significant: improvement in job rewards was not significantly associated with sickness absence |
| Shiri et al. 2024 | Registered sickness absence: at least one episode of illness-related absence lasting more than 10 consecutive workdays – obtained from the employers’ records | effort/reward imbalance | Effort–reward imbalance: ratio of (effort score/reward score) x 0.333 (correction factor) | Non-significant: reduction in effort–reward ratio was not significantly associated with sickness absence |
| Shiri et al. 2024 | Registered sickness absence: at least one episode of illness-related absence lasting more than 10 consecutive workdays – obtained from the employers’ records | job control – work-time control | Work-time control: seven items | Non-significant: improvement in work-time control was not significantly associated with sickness absence |
| Pedersen et al. 2024 | Sickness absence: based on data from the Danish labor market accountant (LMA) and work absences (RoWA) registries | work-related stress | Work-stress: combining three dichotomous (1=yes or 0=no) work-stress indicators – self-perceived work stress, the Cohen four-item perceived stress scale modified to work stress, and job strain (high quantitative demands and low influence/job control at work) (classified as having either zero, one, any combination of two, or all three work-stress indicators) | Significant: having at least 2 of 3 (vs. 0 of 3) work-stress indicators was associated with more days of sickness absence among men; having at least 1 of 3 (vs. 0 of 3) work-stress indicators was associated with more days of sickness absence among women |
| Farrants et al. 2022 | Long-term sickness absence: >183 net days with sickness absence benefits from the Social Insurance Agency | demand/control status | Job demands and job control status (job exposure matrix): psychosocial job exposure matrix (JEM) (Fredlund et al. (2000)) | Non-significant: none of the associations between job demands/job control and long-term sickness absence (>183 days) were statistically significant |
| Wang et al. 2022 | Sickness absence | job demands | Job demand: Swedish psychosocial Job Exposure Matrix (JEM) | Significant: lower job demands were significantly associated with a lower risk of receiving sickness benefits (1–30 days and 31-365 days, all models) |
| Wang et al. 2022 | Sickness absence | job control | Job control: Swedish psychosocial Job Exposure Matrix (JEM) | Significant: higher job control was significantly associated with a lower risk of receiving sickness benefits (1–30 days all but the fully adjusted model / 31-365 days all models) |
| Wang et al. 2022 | Sickness absence | social support – at work | Social support: Swedish psychosocial Job Exposure Matrix (JEM) | Significant: higher social support was significantly associated with a higher risk of receiving sickness benefits (1–30 days all models / 31-365 days all but the fully adjusted model) |
| Pedersen et al. 2021 | Sickness absence due to an individual’s own sickness: short periods of sickness absence from one to thirty days and prolonged sickness absence periods when the employer is compensated by receiving sickness absence benefit | work-related stress | Work-related perceived stress: "How often have you felt stressed in the last two weeks?" (always, often, sometimes, seldom, or never) / "What was the most important source of your stress?" (work, personal life, or work and personal life) – work-related stress ("always or often" responses to the first question, and "work" response to the second question) | Significant: the expected average number of days spent in sickness absence was higher among men and women reporting frequent work-related stress (vs. those with no perceived stress) |
| Oliv et al. 2019 | Sickness absence among workers with neck or upper back pain (self-reported pain in the "upper back or neck" after work during the last 3 months) | job demands | Job demand: work stress, work attention, concentration, work load | Non-significant: job demands were not significantly associated with the number of net sick days among workers with neck or upper back pain |
| Oliv et al. 2019 | Sickness absence among workers with neck or upper back pain (self-reported pain in the "upper back or neck" after work during the last 3 months) | social support – at work | Support: support form supervisors and support from fellow workers | Non-significant: support was not significantly associated with the number of net sick days among workers with neck or upper back pain |
| Oliv et al. 2019 | Sickness absence among workers with neck or upper back pain (self-reported pain in the "upper back or neck" after work during the last 3 months) | job control | Job control: work tempo, work organization, work planning, work influence | Significant: high job control was significantly associated with a lower number of net sick days among workers with neck or upper back pain |
| Bouville et al. 2018 | Sickness absences: the number of self-reported sickness-related absence days within the previous 12 months – report only doctor certified sickness-related absences | job demands | Job demands: nine items from Karasek’s Job Content Questionnaire (JCQ; Karasek et al., 1998 – e.g., "My job requires me working hard") | Significant: high (vs.low) job demands were associated with more days of absence among older workers, while high (vs. low) job demands were associated with a lower number of days of absence among younger workers |
| Bouville et al. 2018 | Sickness absences: the number of self-reported sickness-related absence days within the previous 12 months – report only doctor certified sickness-related absences | skill discretion – skill variety | Skill variety: four items from Karasek’s Job Content Questionnaire (JCQ; Karasek et al., 1998 – e.g., "My job requires me a high skill level") | Significant: high (vs.low) skill variety was associated with more days of absence among older workers, while high (vs. low) skill variety was associated with a lower number of days of absence among younger workers |
| Bouville et al. 2018 | Sickness absences: the number of self-reported sickness-related absence days within the previous 12 months – report only doctor certified sickness-related absences | social support – manager | Supervisor support: four items from Karasek’s Job Content Questionnaire (JCQ; Karasek et al., 1998 – e.g., "My supervisor is concerned with me") | Significant: high (vs.low) supervisor support was associated with more days of absence among older clerks and blue-collar workers, while high (vs. low) supervisor support was associated with a lower number of days of absence among younger clerks and blue-collar workers; Non-significant: supervisor support was not associated with sickness absence among older workers when not stratified by occupational group |
| Bouville et al. 2018 | Sickness absences: the number of self-reported sickness-related absence days within the previous 12 months – report only doctor certified sickness-related absences | social support – coworkers | Colleagues’ support: four items from Karasek’s Job Content Questionnaire (JCQ; Karasek et al., 1998 – e.g., "Colleagues in my job are helpful") | Significant: high (vs. low) colleague support was associated with decreased absences in older clerks and blue-collar workers – significantly sharper dicrease among clerks vs. blue-collar workers |
| Bouville et al. 2018 | Sickness absences: the number of self-reported sickness-related absence days within the previous 12 months – report only doctor certified sickness-related absences | job control – autonomy | Autonomy: four items from Karasek’s Job Content Questionnaire (JCQ; Karasek et al., 1998 – e.g., "My job allows me own decisions") | Significant: older clerks with high (vs. low) autonomy had the fewest absences (not statistically different from younger clerks); older blue-collars were significantly less absent than younger blue-collars when provided with greater (vs. lower) autonomy |
| Götz et al. 2018 | Sickness days: total number of sickness-related days of absence from work – open question on how many days participants were not able to work because of illness in the previous year | work effort | Effort: three items from the short version of the ERI questionnaire – perceived psychological demands at work | Significant: high effort was linked to a higher number of sickness days among women in most models, while among men this association was only significant in multivariable negative-binominal regression analyses |
| Götz et al. 2018 | Sickness days: total number of sickness-related days of absence from work – open question on how many days participants were not able to work because of illness in the previous year | job rewards | Rewards: seven items from the short version of the ERI questionnaire – salary, esteem, job security, and career opportunities | Significant: low reward was linked to a higher number of sickness days among men (except for models adjusting for self-rated health) in multivariable negative-binominal regression analyses; Non-significant: low reward was not significantly associated with the number of sickness days among older women |
| Götz et al. 2018 | Sickness days: total number of sickness-related days of absence from work – open question on how many days participants were not able to work because of illness in the previous year | effort/reward imbalance | Effort–reward imbalance: dividing the sum score of the "effort" items (nominator) through the sum score of the "reward" items (adjusted for number of items; denominator). | Significant: an effort-reward ratio of >1 was linked to a higher number of sickness days among both men (except for models adjusting for self-rated health) and women |
| Sundstrup et al. 2018 | Long-term sickness absence: sickness absence >30 calendar days, corresponding to ≥6 consecutive weeks in DREAM | job demands – quantitative | Quantitative demands: "How often did you not have time to complete all your work tasks?" (COPSOQ) | Non-significant: quantitative demands were not significantly associated with long-term sickness absence |
| Sundstrup et al. 2018 | Long-term sickness absence: sickness absence >30 calendar days, corresponding to ≥6 consecutive weeks in DREAM | job demands – work pace | Work pace: "Did you have to work very fast?" Copenhagen Psychosocial Questionnaire (COPSOQ) | Non-significant: work pace was not significantly associated with long-term sickness absence |
| Sundstrup et al. 2018 | Long-term sickness absence: sickness absence >30 calendar days, corresponding to ≥6 consecutive weeks in DREAM | mentally stimulating or demanding work | Cognitive demands: "Did your work require you to make difficult decisions?" (COPSOQ) | Significant: high (but not medium) cognitive demands were significantly associated with a higher risk of long-term sickness absence |
| Sundstrup et al. 2018 | Long-term sickness absence: sickness absence >30 calendar days, corresponding to ≥6 consecutive weeks in DREAM | job demands – emotional | Emotional demands: "Did you have to relate to other people’s personal problems as part of your work?" (COPSOQ) | Significant: high and medium emotional demands were significantly associated with a higher risk of long-term sickness absence |
| Sundstrup et al. 2018 | Long-term sickness absence: sickness absence >30 calendar days, corresponding to ≥6 consecutive weeks in DREAM | job control – influence | Influence at work: "Did you have a large degree of influence concerning your work?" (COPSOQ) | Significant: low (but not medium – p=0.079) influence at work was significantly associated with a higher risk of long-term sickness absence |
| Sundstrup et al. 2018 | Long-term sickness absence: sickness absence >30 calendar days, corresponding to ≥6 consecutive weeks in DREAM | skill discretion – learning opportunities | Possibilities for development: "Did you have the possibility of learning new things through your work?" (Copenhagen Psychosocial Questionnaire (COPSOQ)) | Non-significant: possibilities for development were not significantly associated with long-term sickness absence |
| Sundstrup et al. 2018 | Long-term sickness absence: sickness absence >30 calendar days, corresponding to ≥6 consecutive weeks in DREAM | recognition | Recognition from management: "Was your work recognized and appreciated by the management?" (Copenhagen Psychosocial Questionnaire (COPSOQ)) | Non-significant: recognition from management was not significantly associated with long-term sickness absence |
| Sundstrup et al. 2018 | Long-term sickness absence: sickness absence >30 calendar days, corresponding to ≥6 consecutive weeks in DREAM | role clarity | Role clarity: "Did you know exactly which areas were your responsibilities?" (COPSOQ) | Significant: medium (but not low) role clarity was significantly associated with a higher risk of long-term sickness absence |
| Sundstrup et al. 2018 | Long-term sickness absence: sickness absence >30 calendar days, corresponding to ≥6 consecutive weeks in DREAM | role conflicts | Role conflicts: "Were contradictory demands placed on you at work?" (COPSOQ) | Significant: high (p=0.007) and medium (p=0.050) role conflicts were significantly associated with a higher risk of long-term sickness absence |
| Sundstrup et al. 2018 | Long-term sickness absence: sickness absence >30 calendar days, corresponding to ≥6 consecutive weeks in DREAM | social support – coworkers | Social support from colleagues: "Did your colleagues talk with you about how well you carry out your work?" (COPSOQ) | Non-significant: social support from colleagues was not significantly associated with long-term sickness absence |
| Sundstrup et al. 2018 | Long-term sickness absence: sickness absence >30 calendar days, corresponding to ≥6 consecutive weeks in DREAM | social support – manager | Social support from supervisors: "Did your nearest superior talk with you about how well you carry out your work?" (COPSOQ) | Non-significant: social support from supervisors was not significantly associated with long-term sickness absence |
| Sundstrup et al. 2018 | Long-term sickness absence: sickness absence >30 calendar days, corresponding to ≥6 consecutive weeks in DREAM | relationships at work | Social community at work: "Was there a good atmosphere between you and your colleagues?" (COPSOQ) | Non-significant: social community at work was not significantly associated with long-term sickness absence |
| Sundstrup et al. 2018 | Long-term sickness absence: sickness absence >30 calendar days, corresponding to ≥6 consecutive weeks in DREAM | job demands | Domain of demands at work: quantitative demands, work pace, emotional demands, cognitive demands | Significant: high (but not medium) domain of demands at work was significantly associated with a higher risk of long-term sickness absence |
| Sundstrup et al. 2018 | Long-term sickness absence: sickness absence >30 calendar days, corresponding to ≥6 consecutive weeks in DREAM | influence and learning opportunities | Domain of work organization and job content: influence, possibilities for development | Significant: poor (but not medium) domain of work organization and job content was significantly associated with a higher risk of long-term sickness absence |
| Sundstrup et al. 2018 | Long-term sickness absence: sickness absence >30 calendar days, corresponding to ≥6 consecutive weeks in DREAM | relationships at work and leadership | Domain of interpersonal relations and leadership: recognition from management, role clarity, role conflicts, social support from colleagues/supervisors, social community at work | Significant: medium (but not poor) domain of interpersonal relations and leadership was significantly associated with a higher risk of long-term sickness absence |
| Sundstrup et al. 2018 | Long-term sickness absence: sickness absence >30 calendar days, corresponding to ≥6 consecutive weeks in DREAM | demand/control status | Demand-job control ratio: dividing the demand domain by the domain of work organization and job content to approximate the job strain model | Non-significant: demand-job control ratio was not significantly associated with long-term sickness absence |
| Sundstrup et al. 2018 | Long-term sickness absence: sickness absence >30 calendar days, corresponding to ≥6 consecutive weeks in DREAM | effort/reward imbalance | Demand-reward ratio: dividing the demand domain by score combining the items of "recognition from management", "social support from co-workers" and "social support from supervisors" to approximate the effort-reward imbalance model | Significant: medium (but not high – p=0.095) demand-reward ratio was significantly associated with a higher risk of long-term sickness absence |
| Tenhiala et al. 2013 | Short (1-3 days) and long (>3 days) spells of sickness absence: number of absences per two years obtained from employers’ registers | organizational justice | Procedural justice scale: respondents' assessment of the formal procedures followed in the workplace, including procedures for collecting accurate information to decision making, providing opportunities to appeal or challenge decisions, generating standards that ensure consistent decisions, and hearing the concerns of all individuals affected by decisions | Significant only for long spells (>3 days) of sickness absence: older employees (over 55 years of age) who perceived a higher level of procedural justice in their work were less likely to be absent from work due to medically certified illnesses |
| Siukola et al. 2011 | Sickness absence days (change measured at baseline and after 4 years) | organizational climate | Team spirit and reactivity: 6 items (e.g., "My colleagues discuss improvements to the work and/or the work environment") – change measured at baseline and after 4 years | Non-significant: an association was observed between change in sickness absence and change in team spirit and reactivity only among the <50 group |
| Siukola et al. 2011 | Sickness absence days (change measured at baseline and after 4 years) | job rewards | Incentive system: 5 items (e.g., "Personnel have an opportunity to develop their own work and work environment in this company") – change measured at baseline and after 4 years | Non-significant: changes in incentive systems were not significantly associated with changes in sickness absence days |
| Siukola et al. 2011 | Sickness absence days (change measured at baseline and after 4 years) | job rewards | Extrinsic incentives: 5 items (e.g., "I get encouraging feedback on my work") – change measured at baseline and after 4 years | Non-significant: changes in extrinsic incentives were not significantly associated with changes in sickness absence days |
| Siukola et al. 2011 | Sickness absence days (change measured at baseline and after 4 years) | leadership | Incentive and participative leadership: 6 items (e.g., "My manager pays attention to my suggestions and wishes") – change measured at baseline and after 4 years | Non-significant: changes in incentive and participative leadership were not significantly associated with changes in sickness absence days |
| Siukola et al. 2011 | Sickness absence days (change measured at baseline and after 4 years) | task and goal system | Task and goal system: 4 items (e.g., "This company has clear and logical/realistic goals") – change measured at baseline and after 4 years | Non-significant: changes in the task and goal system were not significantly associated with changes in sickness absence days |
| Siukola et al. 2011 | Sickness absence days (change measured at baseline and after 4 years) | task value | Task value: 3 items (e.g., "My job includes different and varied tasks") – change measured at baseline and after 4 years | Non-significant: changes in task value were not significantly associated with changes in sickness absence days |
| Siukola et al. 2011 | Sickness absence days (change measured at baseline and after 4 years) | job control – influence | Opportunities to influence one’s work: change measured at baseline and after 4 years | Non-significant: changes in opportunities to influence were not significantly associated with changes in sickness absence days |

**Supplementary Table 3.** Summary of main results by exposure to physical working conditions within each included article

| **Author** | **Outcome definition** | **Exposure** | **Exposure definition** | **Results summary** |
| --- | --- | --- | --- | --- |
| **General physical work demands** | | | | |
| Pedersen et al. 2022 | Sickness absence: periods when the individual is registered as sick-listed by the employer or receiving sickness absence benefit | physically demanding work | Physical work demands score: measured through an ergonomic index, which was constructed by seven questions | Significant: women with moderate and high physical work demands experience an almost identical increase of sickness absence time (+12/+13 days), women with very high physical work demands experience longer sickness absence (+23 days) compared to women with low physical work demands (28 days), high and very high level physical work demands were linked to longer sickness absence among men (+20 days/++12days); Non-significant: moderate physical work demands have no significant effect on men |
| Andersen et al. 2021 | Long-term sickness absence: having registered sickness absence in DREAM for a period of at least six consecutive weeks for a period of up to 2 years starting the week after the questionnaire reply | physically demanding work | Physical work demands: "How much of your working time do you …" (1) walk or stand? (2) work with twisted or bent back without support from the hands and arms? (3) have the arms lifted to or above shoulder height, (4) do the same arm movements several times a minute? (eg, package work, mounting, machine feeding, carving), (5) squat or kneel when you work? (6) push or pull? and (7) lift or carry? | Significant: moderate/high/very high physical work demands were significantly associated with a higher risk of long-term sickness absence (vs. low demands) among the total population, as well as among women and men – with the exception of moderate physical demands, which was not significantly associated with long-term sickness absence among men (only among women) |
| Pedersen et al. 2020 | Sickness absence: receiving sickness absence benefits based on records from the DREAM register | physically demanding work | Physical work demand: linking a job exposure matrix (JEM) to the study population by their age, sex and occupation code – JEM values on the physical work demand were estimated by regression analysis (categorized as low, medium, or high physical demands) | Significant: a 50-year-old woman exposed to high physical work demands was expected to spend 0.43 years more on sickness absence than a woman exposed to low physical work demands; the comparable difference for a 50-year-old man was 0.59 years |
| Sundstrup et al. 2018 | Long-term sickness absence: sickness absence >30 calendar days, corresponding to ≥6 consecutive weeks in DREAM | physically demanding work | Physical work demands: based on a question from the Copenhagen Male Study – "Looking back on your entire working life: For how many years of your working life have you had…, 1) mostly sedentary work without physical strain?, 2) mostly standing or walking work without major physical strain?, 3) mostly standing or walking work with some lifting and carrying?, 4) mostly heavy, fast or physically demanding work?" (categories defined as low, moderate, high, or very high physical work demands) | Significant: moderate, hard, and very hard physical work were significantly associated with a higher risk of long-term sickness absence (vs. sedentary work) – the higher the physical work demand the higher the risk of absence |
| **Ergonomic hazards** | | | | |
| Bláfoss et al. 2023 | Long-term sickness absence: ≥6 weeks of registered sickness absence for a period of ≤2 years | heavy lifting | Lifting duration and the typical load of the lifting tasks during a working day were assessed using two questions: "How much of your working day do you carry or lift?" and "What does what you carry or lift typically weigh?" | Significant: heavy lifting duration (1/2 or 3/4 of the workday vs. seldom) and lifting load (5–15/16–29/≥30kg vs. <5kg) were significantly associated with long-term sickness absence (LTSA); Non-significant: lifting duration of 1/4 of the workday was not significantly associated with LTSA when compared to seldom lifting |
| Oliv et al. 2019 | Sickness absence among workers with neck or upper back pain (self-reported pain in the "upper back or neck" after work during the last 3 months) | heavy lifting | Lifting ≥15 kg | Significant: lifting ≥15 kg was significantly associated with a higher number of net sick days among workers with neck or upper back pain |
| Oliv et al. 2019 | Sickness absence among workers with neck or upper back pain (self-reported pain in the "upper back or neck" after work during the last 3 months) | work posture | Twisted work posture, leaning forward without support, hands at shoulder level or higher | Marginally significant: twisted work posture (p=0.05) and leaning forward without support (p=0.07) was marginally significantly associated with a higher number of net sick days among workers with neck or upper back pain; Non-significant: hands at shoulder level or higher was not significantly associated with the number of net sick days among workers with neck or upper back pain |
| Oliv et al. 2019 | Sickness absence among workers with neck or upper back pain (self-reported pain in the "upper back or neck" after work during the last 3 months) | repetitive movements | Repetitive movements, frequent trunk rotations | Significant: frequent trunk rotations were significantly associated with a higher number of net sick days among workers with neck or upper back pain; Non-significant: repetitive movements were not significantly associated with the number of net sick days among workers with neck or upper back pain |
| Oliv et al. 2019 | Sickness absence among workers with neck or upper back pain (self-reported pain in the "upper back or neck" after work during the last 3 months) | exposure to vibrations | Hand held vibrating tools, whole-body vibrations | Non-significant: hand held vibrating tools and whole-body vibrations were not significantly associated with the number of net sick days among workers with neck or upper back pain |
| Oliv et al. 2019 | Sickness absence among workers with neck or upper back pain (self-reported pain in the "upper back or neck" after work during the last 3 months) | work posture – sitting | Seated work | Significant: seated work was significantly associated with a lower number of net sick days among workers with neck or upper back pain |
| Sundstrup et al. 2018 | Long-term sickness absence: sickness absence >30 calendar days, corresponding to ≥6 consecutive weeks in DREAM | exposure to vibrations | Exposure to vibrations: "In your current or previous job are/were you often exposed to the following in your daily work (several times a week or more)… hand tools vibrations?" | Significant: long-term (≥20 years) vibration exposure (hand tool) was significantly associated with long-term sickness absence |
| Sundstrup et al. 2018 | Long-term sickness absence: sickness absence >30 calendar days, corresponding to ≥6 consecutive weeks in DREAM | heavy lifting | Lifting/carrying of heavy burdens: "In your current or previous job are/were you often exposed to the following in your daily work (several times a week or more)… lift or move heavy things or persons?" | Significant: exposure to lifting/carrying of heavy burdens for <10, <20, or ≥20 years was significantly associated with long-term sickness absence |
| Sundstrup et al. 2018 | Long-term sickness absence: sickness absence >30 calendar days, corresponding to ≥6 consecutive weeks in DREAM | pulling/pushing heavy burdens | Pushing/pulling of heavy burdens: "In your current or previous job are/were you often exposed to the following in your daily work (several times a week or more)… pull or push heavy burdens?" | Significant: short- (<10 years) and long-term (≥20 years) exposure to pushing/pulling of heavy burdens was significantly associated with long-term sickness absence |
| Sundstrup et al. 2018 | Long-term sickness absence: sickness absence >30 calendar days, corresponding to ≥6 consecutive weeks in DREAM | work posture – back twisted/bent | Back twisted/bent: "In your current or previous job are/were you often exposed to the following in your daily work (several times a week or more)… work in stooping posture without leaning on hands or arms? / work in which you have to twist or bend your back several times per hour?" | Significant: long-term (≥20 years) exposure to work in stooping posture, as well as short- (<10 years) and long-term (≥20 years) exposure to frequent back twisting/bending, were significantly associated with long-term sickness absence |
| Sundstrup et al. 2018 | Long-term sickness absence: sickness absence >30 calendar days, corresponding to ≥6 consecutive weeks in DREAM | repetitive movements | Repetitive frequent movements: "In your current or previous job are/were you often exposed to the following in your daily work (several times a week or more)… work where you repeat the same movements several times per minute during a large part of the working hours?" | Significant: exposure to frequent repetitive movements for <10, <20, or ≥20 years was significantly associated with long-term sickness absence when adjusted for age and gender; Non-significant: in the fully adjusted model (age, gender, psychosocial work environment, lifestyle, chronic diseases, socioeconomic position, previous LTSA) |
| Sundstrup et al. 2017 | Long-term sickness absence: sickness absence >30 calendar days, corresponding to ≥6 consecutive weeks in DREAM | heavy lifting | Ton-year: lifting one ton per working day for one year / Frequent heavy lifting-year: lifting loads weighing 20kg ten times per working day for one year (0, >0–<10, 10–<20, ≥20 exposure years) | Significant: heavy lifting (>0–<10, 10–<20, ≥20 years of exposure to ton- or frequent heavy-lifting) was associated with a significantly higher risk of long-term sickness absence – the longer the exposure to heavy lifting the higher the risk of long-term sickness absence |
| Sundstrup et al. 2017 | Long-term sickness absence: sickness absence >30 calendar days, corresponding to ≥6 consecutive weeks in DREAM | work posture – kneeling | Kneeling-year: kneeling for one hour each working day in one year (0, >0–<10, 10–<20, ≥20 exposure years) | Significant: >0–<10 or ≥20 years of exposure to kneeling work posture (but not 10–<20 years) was associated with a significantly higher risk of long-term sickness absence – the longer the exposure to kneeling work posture the higher the risk of long-term sickness absence |
| Sundstrup et al. 2017 | Long-term sickness absence: sickness absence >30 calendar days, corresponding to ≥6 consecutive weeks in DREAM | exposure to vibrations | Whole-body vibration-year: being exposed to whole body vibration for one hour of each working day in one year (0, >0–<10, 10–<20, ≥20 exposure years) | Significant: >0–<10 years of exposure to whole-body vibration (but not 10–<20 or ≥20 years) was associated with a significantly higher risk of long-term sickness absence |
| Siukola et al. 2011 | Sickness absence days (change measured at baseline and after 4 years) | repetitive movements | Repetitive and monotonous movements: change measured at baseline and after 4 years | Non-significant: changes in repetitive and monotonous movements were not significantly associated with changes in sickness absence days |
| Siukola et al. 2011 | Sickness absence days (change measured at baseline and after 4 years) | work posture | Poor working postures: change measured at baseline and after 4 years | Significant: an increased change in poor working postures was associated with an increased change in sickness absence days |
| **Environmental hazards** | | | | |
| Sundstrup et al. 2018 | Long-term sickness absence: sickness absence >30 calendar days, corresponding to ≥6 consecutive weeks in DREAM | exposure to noise | Exposure to noise: "In your current or previous job are/were you often exposed to the following in your daily work (several times a week or more)… noise so loud that you must raise your voice to talk to other people?" | Significant: short- (<10 years) and long-term (≥20 years) noise exposure was significantly associated with long-term sickness absence |
| Sundstrup et al. 2018 | Long-term sickness absence: sickness absence >30 calendar days, corresponding to ≥6 consecutive weeks in DREAM | exposure to dust | Exposure to dust: "In your current or previous job are/were you often exposed to the following in your daily work (several times a week or more)… dust? (cement, demolitions, mineral fibers, wood, animals or plants)" | Significant: long-term (≥20 years) exposure to dust was significantly associated with long-term sickness absence |
| Sundstrup et al. 2018 | Long-term sickness absence: sickness absence >30 calendar days, corresponding to ≥6 consecutive weeks in DREAM | exposure to toxic substances | Exposure to toxic substances: "In your current or previous job are/were you often exposed to the following in your daily work (several times a week or more)… toxic substances?" | Significant: exposure to toxic substances for <10, <20, or ≥20 years was significantly associated with long-term sickness absence when adjusted for age and gender; Non-significant: in the fully adjusted model (age, gender, psychosocial work environment, lifestyle, chronic diseases, socioeconomic position, previous LTSA) |
| Sundstrup et al. 2018 | Long-term sickness absence: sickness absence >30 calendar days, corresponding to ≥6 consecutive weeks in DREAM | exposure to welding smoke | Exposure to welding smoke: "In your current or previous job are/were you often exposed to the following in your daily work (several times a week or more)… welding smoke?" | Significant: exposure to welding smoke for <20 years was significantly associated with long-term sickness absence when adjusted for age and gender; Non-significant: in the fully adjusted model (age, gender, psychosocial work environment, lifestyle, chronic diseases, socioeconomic position, previous LTSA) |
| Sundstrup et al. 2018 | Long-term sickness absence: sickness absence >30 calendar days, corresponding to ≥6 consecutive weeks in DREAM | exposure to diesel fumes | Exposure to diesel fumes: "In your current or previous job are/were you often exposed to the following in your daily work (several times a week or more)… diesel fumes?" | Significant: exposure to diesel fumes for <20 or ≥20 years was significantly associated with long-term sickness absence when adjusted for age and gender; Non-significant: in the fully adjusted model (age, gender, psychosocial work environment, lifestyle, chronic diseases, socioeconomic position, previous LTSA) |
| Siukola et al. 2011 | Sickness absence days (change measured at baseline and after 4 years) | environmental exposure | Environmental exposure (draught, noise, indoor climate, lighting, heat, and cold): changes measured at baseline and after 4 years | Non-significant: changes in environmental exposures were not significantly associated with changes in sickness absence days |
| Preziosi et al. 2004 | Self-reported sickness absence | heating, ventilation, and air-conditioning | Heating, ventilation, and air-conditioning (HVAC): type of ventilation – "Is air-conditioning in use in your workplace?" | Significant: being exposed to heating, ventilation, and air-conditioning systems (vs. natural ventilation) was associated with a higher odds of sickness absence |

**Supplementary Table 4.** Summary of main results by work time-related exposure within each included article

| **Author** | **Outcome definition** | **Exposure** | **Exposure definition** | **Results summary** |
| --- | --- | --- | --- | --- |
| Piszczek et al. 2021 | Sick days: self-reported count of the number taken in the previous year | work-time flexibility | Flexible work hours: "You have flexible working hours" | Significant: flexible work hours were significantly associated with lower sick day use among older employees – the marginal effects of flexible work hours on sick day use were statistically significant at ages 51 and above (non-significant at the age of 50) |
| Bernstrøm and Houkes 2020 | Short- (1-8 days) and long-term (≥9 days) sickness absence: hospitals HR registry of all employee absence | shift work | Day-evening rotations | Significant: day-evening rotations were significantly associated with higher probabilities of both short-term and long-term sickness absence among employees aged 50 and 60 years compared with fixed day shifts |
| Bernstrøm and Houkes 2020 | Short- (1-8 days) and long-term (≥9 days) sickness absence: hospitals HR registry of all employee absence | shift work | Day-evening-night rotations | Significant: day-evening-night rotations were significantly associated with higher probabilities of both short-term and long-term sickness absence among employees aged 50 and 60 years compared with fixed day shifts |
| Larsen et al. 2020 | Long-term sickness absence: ≥30 consecutive days of sickness absence – retrieved from the Danish Working Hour Database and the Working Hours in the Finnish Public Sector Study database | long working hours and shift work | Working hours and shift work: the amount of day shifts (≥3 h between 06:00 and <21:00), evening shifts (≥3 h between 18:00 and <02:00), night shifts (≥3 h between 23:00 and 06:00), long shifts (9 to <12 h), very long shifts (12 to <24 h), quick returns (<11 h between shifts), long work weeks (>40 h/week), very long work weeks (>48 h/week), and periods of consecutive night shifts (≥5) per year | Denmark:  Significant: A higher number of evening and night shifts (>50 per year), very long work weeks (1–12 per year), and consecutive night shifts (≥5) were associated with an increased risk of first-time long-term sickness absence, whereas a higher number of day shifts (>100 per year) was associated with a lower risk; Non-significant: The incidence of first-time long-term sickness absence was not significantly associated with the number of long shifts, very long shifts, quick returns, or long work weeks. |
| Larsen et al. 2020 | Long-term sickness absence: ≥30 consecutive days of sickness absence – retrieved from the Danish Working Hour Database and the Working Hours in the Finnish Public Sector Study database | long working hours and shift work | Working hours and shift work: the amount of day shifts (≥3 h between 06:00 and <21:00), evening shifts (≥3 h between 18:00 and <02:00), night shifts (≥3 h between 23:00 and 06:00), long shifts (9 to <12 h), very long shifts (12 to <24 h), quick returns (<11 h between shifts), long work weeks (>40 h/week), very long work weeks (>48 h/week), and periods of consecutive night shifts (≥5) per year | Finland:  Significant: A higher number of long shifts (>50 per year) and long work weeks (1–50 per year) were associated with an increased risk of first-time long-term sickness absence; Non-significant: The incidence of first-time long-term sickness absence was not significantly associated with the number of day, evening, or night shifts, very long shifts, quick returns, very long work weeks, or consecutive night shifts |
| Ropponen et al. 2020 | Short-term sickness absence: at least one 1-3-day long sickness absence – retrieved from the Finnish Public Sector study | long working hours | Working hours: daily and weekly working hours, the proportion of work weeks exceeding 40 and 48 hours, and shifts longer than 12 hours | Significant: higher weekly working hours and a greater proportion of work weeks exceeding 40 hours were associated with an increased risk of short-term sickness absence; Non-significant: short-term sickness absence was not significantly associated with daily working hours, the proportion of work weeks exceeding 48 hours, and shifts longer than 12 hours |
| Ropponen et al. 2020 | Short-term sickness absence: at least one 1-3-day long sickness absence – retrieved from the Finnish Public Sector study | shift work | Time of day: the proportion of morning, day, evening, and night shifts | Significant: higher percentage of morning shifts were associated with an increased risk of short-term sickness absence; Non-significant: short-term sickness absence was not significantly associated the proportion of day, evening, or night shifts |
| Ropponen et al. 2020 | Short-term sickness absence: at least one 1-3-day long sickness absence – retrieved from the Finnish Public Sector study | shift intensity | Shift intensity: quick returns, number of consecutive shifts, and consecutive evening and night shifts of at least two shifts | Non-significant: short-term sickness absence was not significantly associated with quick returns, consecutive shifts, or consecutive evening or night shifts |
| Afsa and Givord 2014 | Sickness absence of 1 week or more: not having worked due to illness during the entire "reference week," that is the calendar week before the interview (approved by a physician) | irregular schedule | Working time arrangement: working irregular schedules (i.e., with schedules varying from week to week) | Non-significant: working irregular schedules (vs. regular schedules) was not significantly associated with a higher probability of being absent for sickness reasons among older workers |
| Afsa and Givord 2014 | Sickness absence of 1 week or more: not having worked due to illness during the entire "reference week," that is the calendar week before the interview (approved by a physician) | 24-hour shift work | Working time arrangement: working around the clock (usually in a three shifts) | Non-significant: 24-hour shift work (vs. regular schedules) was not significantly associated with a higher probability of being absent for sickness reasons among older workers |
